# Deoxyuridines from the Marine Sponge Associated Actinomycete *Streptomyces microflavus*

**DOI:** 10.3390/md9050690

**Published:** 2011-04-26

**Authors:** Ke Li, Qiao-Lian Li, Nai-Yun Ji, Bo Liu, Wei Zhang, Xu-Peng Cao

**Affiliations:** 1 Dalian Institute of Chemical Physics, Chinese Academy of Sciences, Dalian 116023, China; 2 Liaoning Norm University, Dalian 116029, China; E-Mail: liqiaolian0202@yahoo.com.cn; 3 Yantai Institute of Coastal Zone Research, Chinese Academy of Sciences, Yantai 264003, China; E-Mail: nyji@yic.ac.cn; 4 Key Laboratory of Marine Bio-resources Sustainable Utilization in Liaoning Province’s University, Dalian Fisheries University, Dalian 116023, China; E-Mail: iocas_liubo@hotmail.com; 5 Flinders Centre for Marine Bioprocessing and Bioproducts, School of Medicine, Flinders University, Adelaide, SA 5042, Australia; E-Mail: wei.zhang@flinders.edu.au

**Keywords:** actinomycete, *Streptomyces microflavus*, *Hymeniacidon perlevis*, deoxyuridine

## Abstract

One new nucleoside derivative, named 3-acetyl-5-methyl-2′-deoxyuridine (**1**), along with two known compounds 3,5-dimethyl-2′-deoxyuridine (**2**) and 3-methyl-2′-deoxyuridine (**3**), were isolated from the cultures of *Streptomyces microflavus*. This strain was an associated actinomycete isolated from the marine sponge *Hymeniacidon perlevis* collected from the coast of Dalian (China). Their structures were elucidated by detailed NMR and MS spectroscopic analysis as well as comparison with literature data.

## 1. Introduction

The marine environment, particularly with sponges, is a rich source of new bioactive metabolites—287 novel metabolites were isolated from marine sponges in 2008 [1]. The availability of biomass is a limiting factor for isolating marine natural products. The widespread isolation of typical microbial metabolites from sponges leads to the hypothesis that these metabolites are in fact the products of microbial metabolism [2]. The isolation of secondary metabolite-producing bacteria from sponges and of microbial secondary metabolism gene clusters from the metagenome of sponges has led to the general understanding that these metabolites are, in many cases, the products of microbial symbionts and are not derived from the microbial diet of sponges [3]. Thus, marine organism-associated microbes have been attracting increasing interest as potential sources of marine natural products in order to solve the supply shortage.

A number of reports have been published on the isolation of actinobacteria from marine organisms [4]. Screening bioactive substances from these marine-derived actinobacteria has yielded several new bioactive metabolites [5–8]. In our recent screening for new natural products, one novel deoxyuridine (**1**), along with two known nucleoside derivatives (**2**, **3**), were isolated from *Streptomyces microflavus* strain No. HVG29 belonging to actinomycetes. To date, there have been no reports of deoxyuridine structures isolated from this species. We report herein the isolation and structure elucidation of **1–3**.

## 2. Results and Discussion

**Figure 1.**
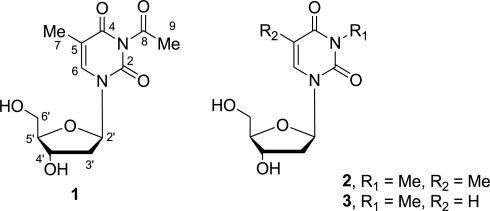
Structures of compounds **1–3**.

The total ethyl acetate extract from 30 L of fermentation broth was partitioned between hexane and 9:1 MeOH–H_2_O followed by diluting the aqueous layer to 3:2 MeOH–H_2_O and solvent partitioning with EtOAc. The EtOAc fraction was subjected to further purification by gel permeation on Sephadex LH-20 to yield compounds **1–3** (Figure 1).

Compound **1** was obtained as a white powder, [*α*]^20^_D_ +5.4° (*c* 0.39, MeOH). The IR spectrum possessed absorptions for hydroxyl groups (3394 cm^−1^) and amide carbonyl groups (1693 cm^−1^). The EIMS gave a molecular ion peak at *m/z* 284 [M]^+^, and the molecular formula C_12_H_16_N_2_O_6_ was determined by HRESIMS at *m/z* 307.0905 [M + Na]^+^ (calcd. for C_12_H_16_N_2_O_6_Na, 307.0906).

**Figure 2.**
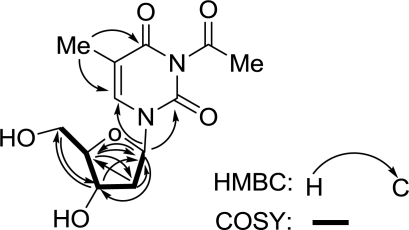
HMBC and ^1^H-^1^H COSY correlations of compound **1**.

In the 1D NMR spectra, proton signals at *δ*_H_ 6.28 (1H, t, *J* = 7.0 Hz, H-2′), 2.22 (2H, m, H-3′), 3.90 (1H, q, *J* = 3.5 Hz, H-4′), 4.40 (1H, dt, *J* = 3.5, 5.9 Hz, H-5′), 3.79 (1H, dd, *J* = 3.2, 12.0 Hz, H-6′a), and 3.83 (1H, dd, *J* = 3.7, 12.0 Hz, H-6′b) and carbon resonances at *δ*_C_ 86.3 (CH, C-2′), 41.2 (CH_2_, C-3′), 72.2 (CH, C-4′), 88.9 (CH, C-5′), and 62.9 (CH_2_, C-6′) supported the presence of a pentose moiety in the molecule. The HMBC spectrum showed correlations from H-6′ to C-4′, from H-5′ to C-2′ and C-3′, from H-4′ to C-2′, from H-3′ to C-5′and C-2′, and from H-2′ to C-5′ and C-4′ (Figure 2). In addition, the correlations between H-2′/H_2_-3′, H_2_-3′/H-4′, H-4′/H-5′, and H-5′/H_2_-6′ were observed in the ^1^H-^1^H COSY spectrum (Figure 2). The above NMR evidence established the presence of a 2′-deoxyribose moiety, which was supported by the similarity of the ^1^H and ^13^C NMR data to those reported in the literatures for such a unit [9,10].

The remaining portion of **1** implied the elemental composition C_7_H_7_N_2_O_3_, and accounted for the remaining 5 degrees of unsaturation. In the 1D NMR spectra, proton signals at *δ*_H_ 7.81 (1H, s, H-6) and 1.88 (3H, s, H-7) and carbon resonances at *δ*_C_ 152.4 (C-2), 166.4 (C-4), 111.5(C-5), 138.2 (C-6), and 12.4 (C-7) evidenced the presence of a thymine moiety in the molecule. An acetyl group was supported by proton signal at *δ*_H_ 1.88 (3H, s, H-9) and carbon signals at *δ*_C_ 23.4 (C-9) and 179.2 (C-8). In combination with the chemical shifts of the corresponding carbons and the elemental composition, the acetyl group was assigned to N-3 of the thymine moiety, because only N-3 had a vacancy in the molecule. Meanwhile, HMBC long-range correlations from H-2′ to C-2 and C-6 (Figure 2), clearly located the 2′-desoxyribose moiety to N-1 of the 3-acetyl-5-methylthymine unit. Therefore, the structure of **1** was determined as 3-acetyl-5-methyl-2′-deoxyuridine.

The structures of known compounds **2** and **3** were confirmed by detailed NMR data comparison with those in literatures [11]. To the best of our knowledge, compound **1** is the first example of acetyl deoxyuridine from marine-derived actinomycetes to be isolated from the marine sponge *Hymeniacidon perlevis*.

## 3. Experimental Section

### 3.1. General

NMR spectra were recorded at 500 and 125 MHz for ^1^H and ^13^C, respectively, on a Bruker Avance 500 NMR spectrometer in acetone-*d*_6_ with TMS as internal standard. Low and high resolution mass spectra were determined on Autospec Premier P776 and VG AutoSpec 3000 mass spectrometers. IR spectra were obtained on a JASCO FT/IR-4100 fourier transform infrared spectrometer. Column chromatography was performed with silica gel (200–300 mesh, Qingdao Haiyang Chemical Co., Qingdao, China) and Sephadex LH-20 (Pharmacia). TLC was carried out with precoated silica gel plates (GF-254, Qingdao Haiyang Chemical Co., Qingdao, China). All solvents were of analytical grade.

### 3.2. Microorganism Material

The sponge-associated actinobacterium *Streptomyces microflavus* was isolated from the inner tissue of the marine sponge *Hymeniacidon perlevis* collected from the inter-tidal beach of the Yellow Sea at Dalian, China (38°52′N, 121°41′E) in March 2003. Sponge specimens were placed in plastic bags containing seawater and immediately transported to the laboratory. Actinobacteria identification was carried out by the method reported by Zhang *et al.* [12]. The sequence data derived from the strain have been submitted and deposited at GenBank with accession number EU554304. BLAST search result showed that the sequence was similar (99%) to the sequence of *S. microflavus* (compared to EU273548). The strain is preserved within the biological technology department, Dalian Institute of Chemical Physics, Chinese Academy of Sciences.

### 3.3. Extraction and Isolation

For chemical investigations, the actinobacterium strain was cultivated statically in special media, which was reported by Xin *et al.* [13]. Mycelium and culture broth of *S. microflavus* (10 L) were homogenized and exhaustively extracted with MeOH and EtOAc, resp. Since the TLC and HPLC profiles of the two extracts were nearly identical, they were combined before further separation. The combined extracts (20 g) were subjected to column chromatography (CC) over SiO_2_ (200–300 mesh) and eluted with different solvents of increasing polarity to yield 11 fractions (Fr. 1–Fr. 11) on the basis of TLC analysis. Fr. 8 was further fractionated by CC on Sephadex LH-20 (CHCl_3_/MeOH, 2:1) and then purified by CC on Sephadex LH-20 (MeOH) to give compound **1** (4.0 mg). Fr. 11 was subjected to CC over SiO_2_ and then further purified by CC on Sephadex LH-20 (CHCl_3_/MeOH, 2:1) to yield compounds **2** (10.8 mg) and **3** (7.0 mg).

3-Acetyl-5-methyl-2′-deoxyuridine (**1**). White powder; [*α*]^20^_D_ +5.4° (*c* 0.39, MeOH); UV (MeOH) λ_max_ 264 (log ε) (3.52), λ_max_ 256 (log ε) (3.46); ^1^H NMR (acetone-*d*_6_, 500 MHz) δ 7.81 (s, H-6), 6.28 (t, *J* = 7.0 Hz, H-2′), 4.40 (dt, *J* = 3.5, 5.9 Hz, H-5′), 3.90 (q, *J* = 3.5 Hz, H-4′), 3.83 (dd, *J* = 3.7, 12.0 Hz, H-6′b), 3.79 (dd, *J* = 3.2, 12.0 Hz, H-6a), 2.22 (m, H-3′), 1.88 (s, H-9), 1.88 (s, H-7); ^13^C NMR (acetone-*d*_6_, 125 MHz) δ 179.2 (C-8), 166.4 (C-4), 152.4 (C-2), 138.2 (C-6), 111.5 (C-5), 88.9 (C-5′), 86.3 (C-2′), 72.2 (C-4′), 62.9 (C-6′), 41.2 (C-3′), 23.4 (C-9), 12.4 (C-7). EIMS *m*/*z* (relative intensity): *m/z* 284 (5), 256 (9), 241 (5), 239 (9), 213 (15), 206 (9), 163 (11), 98 (43), 96 (80), 95 (74), 60 (100); HRESIMS *m/z*: 307.0905 [M + Na]^+^, calcd. for C_12_H_16_N_2_O_6_Na, 307.0906.

## 4. Conclusions

In previous research into metabolites isolated from *S. microflavus*, many fattiviracin derivatives and actinomycin X2 have been elucidated [14]. The major product of these fattiviracin derivatives was fattiviracin FV-8, which consists of four glucose and two trihydroxy fatty acid residues [15–17]. Actinomycin X2 included a wide range of fatty acids (C10–C22), which have been isolated from *Streptomyces nasri* [18]. Compounds **1–3** represent the first examples of deoxyuridine structures isolated from *S. microflavus* which was associated with sponges.

The known compounds (**2** and **3**) were originally isolated from a marine sponge [11], *Geodia barretti*, and now, for the first time, from sponge-associated microorganisms. This finding strengthens the hypothesis that marine microbial symbionts are possibly the true producers or take part in the biosynthesis of some bioactive marine natural products isolated from the marine organism hosts.

These nucleosides may have potent biological or physiological effects. A series of nucleoside analogues were synthesized and exhibited potential antiviral activities against duck hepatitis B virus (DHBV) [19], herpes simplex virus type 1 and 2 (HSV-1 and HSV-2), and varicella zoster virus (VZV) [20–22]. All of those synthesized 2′-deoxyuridine analogues have special substituents on position C-5 of uridine moiety. However, there is no evaluation for the compound which has the substituents on position N-3 of uridine residue. Compounds **1** and **2** provide new thoughts of introducing functional groups on nucleoside structures for synthetic medical chemistry.
